# The Cost of Enfortumab Vedotin Wastage Due to Vial Size—A Real-World Analysis

**DOI:** 10.3390/cancers13235977

**Published:** 2021-11-27

**Authors:** Michal Sarfaty, Assaf Moore, Ashley M. Regazzi, Aaron P. Mitchell, Jonathan E. Rosenberg

**Affiliations:** 1 Genitourinary Oncology Service, Department of Medicine, Memorial Sloan Kettering Cancer Center, New York, NY 10065, USA; regazzia@mskcc.org (A.M.R.); mitchea2@mskcc.org (A.P.M.); rosenbj1@mskcc.org (J.E.R.); 2 Institute of Oncology, Sheba Medical Center, Tel-Hashomer, Ramat Gan 52621, Israel; 3 Oncology Department, Sackler Faculty of Medicine, Tel-Aviv University, Tel-Aviv 69978, Israel; moorea4@mskcc.org; 4 Department of Radiation Oncology, Memorial Sloan Kettering Cancer Center, New York, NY 10065, USA; 5 Oncology Department, Davidoff Cancer Center, Petah-Tikva 41492, Israel; 6 Department of Epidemiology and Biostatistics, Memorial Sloan Kettering Cancer Center, New York, NY 10065, USA; 7 Division of Hematology and Medical Oncology, Weill Cornell Medical College, Cornell University, New York, NY 10021, USA

**Keywords:** enfortumab vedotin, urothelial cancer, drug wastage, vial size

## Abstract

**Simple Summary:**

Enfortumab Vedotin (EV) is FDA-approved for advanced urothelial cancer in patients previously treated with chemotherapy and immunotherapy. In this report, we looked at the extent of EV wastage (i.e., discarding of leftover drug not administered to the patient) in a single institute and estimated the financial impact of EV wastage annually in the United States. We found that wastage occurred in 46% of administered doses, with an average waste per dose of 2.9% (range 0–18%). The average drug wastage cost per patient was $3127 ($252 per dose). The annual cost of EV wastage in the US is estimated to be $15 million.

**Abstract:**

Enfortumab Vedotin (EV) is FDA-approved for advanced urothelial cancer in patients previously treated with platinum-based chemotherapy and a checkpoint inhibitor. We conducted a real-world study to determine the extent of EV wastage in a single institution and assessed the financial impact of EV wastage annually in the United States. Systematic examination of the usage and wastage of all standard-of-care EV treatments administered to urothelial cancer patients at Memorial Sloan Kettering Cancer Center (MSKCC) between 1 January 2020 and 31 December 2020 was performed. Drug wastage was calculated by subtracting the actual administered dose from the total dose in an optimal set of vials. We built a pharmacoeconomic model to assess the financial impact of EV wastage annually in the US using the January 2021 Average Sales Prices from the Centers for Medicare and Medicaid Services. Sixty-four patients were treated with standard-of-care EV, with a median of 11 doses per patient (range 1–28). Wastage occurred in 46% of administered doses (367/793), with a mean waste per dose of 2.9% (0–18%). The average drug wastage cost per patient was $3127 ($252/dose). The annual cost of EV wastage in the US is estimated to be $15 million based on wastage data from a single center in the US. In summary, EV wastage due to available vial sizes was 2.9%, which falls under acceptable thresholds. While the percentage of EV wastage is relatively low, waste-minimizing practices may reduce the financial toxicity for the individual patient and for society.

## 1. Introduction

The ongoing rise in cancer drug prices constitutes a financial burden for both patients and payers throughout the world. Many strategies to lower drug costs have previously been described [1]. One approach to decrease drug-related costs without adversely affecting efficacy is minimizing drug wastage. Drug wastage occurs when a parenteral drug is supplied in fixed-dose vials, and the dose required for an individual patient does not match an amount achievable through any combination of whole vials, resulting in one or more vials being opened but only partially used. Prior studies have estimated wastage to be in the range of 1% to 33% for cancer drugs, with an annual total US revenue of $1.8 billion from discarded drugs [2,3]. Bach, Saltz, and colleagues recommend regulations requiring manufacturers to offer a reasonable selection of vial sizes with an acceptable threshold for vial waste of 3% [2].

Urothelial carcinoma (UC) is the 6th most common cancer, and the second most common cancer of the genitourinary tract. Over 80,000 people are diagnosed with UC in the USA every year, and approximately 18,000 people die of the disease annually. Risk factors of UC include male sex, history of smoking, and advanced age. The median age at diagnosis is 73 years. The prognosis of metastatic UC is poor, with a 5- year survival rate of 5% [4].

Antibody-drug conjugates (ADC) represent a novel form of drug delivery based on a covalent link between a highly specificity monoclonal antibody to a highly active cytotoxic agent [5]. The monoclonal antibody binds to its tumor-associated antigen which induces endocytosis and internalization of the cytotoxic drug. A high affinity and specificity towards well-defined targets on cancer cells enables this technology to expand the therapeutic window of highly toxic active chemotherapy drugs.

Enfortumab Vedotin (EV) is an antibody–drug conjugate that delivers monomethyl auristatin E, a tubulin-disrupting antimitotic agent, to tumors expressing nectin-4. Nectin-4 is a transmembrane protein involved in cellular adhesion abundant on urothelial cancer cells [5]. EV was FDA approved in December 2019 [6] for the treatment of locally advanced or metastatic UCpatients previously treated with platinum-based chemotherapy and a checkpoint inhibitor (CPI). Treatment is administered as an intravenous infusion on days 1, 8, and 15 of a 28-day cycle until disease progression or unacceptable toxicity [7]. The drug is available in 20 mg and 30 mg vials. Initial dose is 1.25 mg/kg per treatment, capped at 125 mg, and dose may be reduced to 1 mg/kg, 0.75 mg/kg, or 0.5 mg/kg in case of toxicity. The combination of EV with pembrolizumab showed promising results in the 1st-line setting in the EV-103 phase I trial [8] and is currently being evaluated in this setting in a phase III trial versus standard chemotherapy. EV is also being assessed in multiple clinical trials in the 1st line and neoadjuvant settings for UC, as well as in other malignancies [9].

The objective of this study is to determine the extent of EV wastage in a single institute, with a correlated pharmacoeconomic model analysis to assess the financial impact of the annual EV wastage in the United States in the third-line setting. The annual EV wastage in the first line setting was also estimated, as EV may be granted approval in this setting in the future.

## 2. Materials and Methods

The study was approved by the Memorial Sloan Kettering Cancer Center (MSKCC) institutional review board prior to data acquisition. A systematic examination of the usage and wastage of all standard-of-care EV treatment dosages administered to UC patients at MSKCC starting EV treatment between 1 January 2020 and 31 December 2020 was performed.

### 2.1. Drug Used and Calculated Waste

For each dose of drug administered, the patient’s weight, planned dose (1.25, 1, 0.75 or 0.5 mg/kg), and actual dose administered (after rounding) were collected. The ideal dose was calculated per patient, defined as

planned dose (mg/kg) × patient’s weight (kg).(1)

The optimal composition of vials per administered dose was determined using 20 mg and 30 mg vials. At MSKCC, any drug remaining in the vial after preparing a patient’s dose must be discarded. Wastage per administration was calculated by subtracting the actual administered dose from the total dose in an optimal set of vials. The proportion of drug wastage was defined as waste divided by the total dose in an optimal set of vials.

### 2.2. Rounding

Most institutions allow for dose rounding of 5–10% [10,11] to minimize waste, as per dosing guidelines. Rounding is used to the nearest 5 mg dose (example: 78, 79, 81, and 82 mg would be rounded to 80 mg, whereas 83, 84, 86, and 87 mg would be rounded to 85 mg). At low total doses (total dose <50 mg), no rounding is done. We calculated waste with and without rounding to assess whether rounding reduces waste-related costs.

### 2.3. Annual Cost of EV Wastage in the US

We performed a budget impact analysis to assess the financial impact of EV wastage from the societal perspective of payers in the United States. EV cost was based on the January 2021 Average Sales Prices from the Centers for Medicare and Medicaid Services ($111.8 per mg) [4]. The price of 1 mg of EV is identical between 20 mg and 30 mg vials. To estimate the annual number of patients treated with EV per FDA approval, we used the estimated UC deaths in 2020 per the Surveillance Epidemiology and End Results Program (SEER) database [12] (17,980 estimated UC deaths in 2020). We then estimated that patients receiving ≥3rd-line treatment would be equivalent to 30% of UC deaths. This estimation took into account a possible overestimation in clinical trial data, reporting that 35–55% [13,14] of trial patients who progressed after CPI received subsequent treatment. Conversely, we appreciated the potential underestimation of published real-world Medicare-based studies [15,16] showing only 8–15% of advanced UC patients receive advanced lines of treatment, as these only included patients over the age of 65 and were collected in the pre-immunotherapy era where 2nd-line treatment was relatively more toxic and less effective. Therefore, we opted to use a range of 15–50% for the sensitivity analysis. We then multiplied the number of EV-treated patients by the average cost of wastage per dose and by the median number of doses per real-world MSKCC data. All variables are provided in Table 1.

### 2.4. Sensitivity Analysis

We performed a sensitivity analysis using a specific range for each base case parameter. All sensitivity analysis ranges are provided in Table 2.

### 2.5. Structural Sensitivity Analysis—EV as 1st Line

We estimated the cost of wastage if EV were to be granted approval for 1st-line treatment in combination with pembrolizumab as tested in the EV-103 trial [8]. In the trial, EV was given on days 1 and 8 of a 21 day cycle. The number of newly diagnosed metastatic UC patients per year was estimated as 20% more than the number UC deaths in 2020 per SEER database [12]. As the combination of EV and pembrolizumab is suitable for both cisplatin eligible and ineligible patients, we estimated that 80% of newly diagnosed metastatic UC patients would receive this combination as 1st line treatment once approved. The estimated median number of doses was based on the median number of cycles in the preliminary results of the EV-103 trial (9 cycles of d1,8 q21d, 18 doses per patient) [8]. All sensitivity analysis ranges are provided in Table 3.

## 3. Results

Between January 2020 to December 2020, 64 patients were treated with EV at MSKCC per standard-of care. A total of 793 doses were administered, median of 11 doses per patient (range 1–28).

Wastage occurred in 367 doses (46%), and the most commonly discarded amount per dose was 5 mg (in 329 doses, 41.5% from total). Across all patients, a total of 61,960 mg was used, of which 60,170 mg of drug was administered to patients and 1790 mg was discarded. Mean waste per dose was 2.9% (range 0–18%), median 2.6% (interquartile range 0–5%), with a total waste across all doses (total mg discarded/total mg of all vials used) of 2.9%. The average drug wastage cost per dose was $252, $3127 per patient. Total wastage cost at MSKCC in 2020 was $200,122 (Table 1).

The estimated annual cost of EV wastage for US payers in 2020 is $14,952,168. Sensitivity analysis is shown in Table 2.

### Structural Sensitivity Analysis—EV as 1st Line

Estimated annual cost of EV wastage as 1st line treatment for US payers in 2020 is $78,294,988 (Table 3).

## 4. Discussion

EV is the first antibody–drug conjugate approved in advanced UC, based on a significant survival benefit as compared with standard chemotherapy in a phase III trial. The monthly cost of EV for an average weight male [17] is over $40K, or $500K per year.

This study analyzed real-world data of standard-of-care usage and wastage of EV in 2020 from a major tertiary cancer center in the US following the drug’s FDA approval in late 2019. EV wastage was found to be 2.9%, less than previously reported on other high-priced drugs in oncology and just under the acceptable threshold suggested by Bach, Saltz, and colleagues [2]. While the percentage of waste for EV is relatively low, its high total cost may still be a significant financial burden on the individual patient, payers, the healthcare system, and society. We estimate that if EV is approved as 1st-line treatment in combination with pembrolizumab, the annual cost of EV wastage in the US will reach $78 million, fivefold compared with the cost of 3rd-line EV. A correlation between patients’ financial toxicity and bankruptcy, delays in care, reduced compliance, and diminished quality of life has been demonstrated in several studies, including in bladder cancer [18,19,20]. Thus, efforts to reduce costs including waste-related to a minimum are necessary.

Several methods to reduce drug wastage have been suggested [21] and some have been implemented to varying degrees in different cancer centers. The Committee on the Implications of Discarded Weight-Based Drugs has recently published its recommendations regarding cost, safety, and quality concerns associated with discarded drugs that result from weight-based dosing of medicines contained in single-dose vials [22]. In their report, they concluded that in the US there is no economic incentive to reduce drug wastage under the current health care reimbursement policy, as health care providers receive reimbursement for the full vial size used and not per dose delivered.

From the individual patient’s perspective, the financial burden of out-of-pocket expenses based on the price of the full vial size may be significant, especially in disadvantaged populations. In several countries outside of the US, EV is not reimbursed due to its high cost, leading some patients to pay the full cost of the drug out-of-pocket. In these selected cases, it may be reasonable to implement cost-saving strategies that are unlikely to adversely impact clinical outcomes.

Rounding of the administered dose is a common practice that simplifies drug preparation and may reduce drug wastage. For antibody–drug conjugates, rounding within 10% of the prescribed dose is considered acceptable and expected not to influence patient outcomes [11]. As seen in our study, rounding to the nearest 5 mg or 10 mg is commonly used, thus developing a smaller size vial of 5 mg by the pharmaceutical company would solve most of the wastage seen with EV. As EV is currently only available in 20 mg and 30 mg vials, a relevant cost-saving strategy may be rounding to the nearest 10 mg dose of each dose, without changing the total dose per cycle. For example, a patient weighing 75 kg will be prescribed an EV dose of 75 kg × 1.25 mg/kg = 94 mg d1, 8, 21 q28d. The treatment dose will likely be rounded to 95 mg per dose, leading to wastage of 5 mg per dose, or 15 mg per cycle, at a cost of $1650 (15 mg × $110 per mg). The same dose per cycle (95 mg × 3 = 280 mg) could be given as 100 mg on d1, and 90 mg on days 8, 21, with no wastage, and still within the constraints of rounding up to 10% of the total dose.

Another cost-saving strategy is vial sharing, meaning a single vial would be used by more than one patient to prevent wastage. In the US, this strategy is not widely accepted, as the CDC issued a warning for the risk of contamination [23]. Arguably, in some countries where economic incentives for minimizing drug waste exist, well-established practices for vial sharing are commonly and safely used [24]. As EV is a new drug with a relatively low number of patients treated per center, vial sharing seems less practical as it would require treating several patients on the same day. This strategy may become more relevant if EV will be approved in the first line setting.

Drug wastage is commonly overlooked when estimating drug costs as part of the incremental cost-effectiveness ratio in cost-effectiveness analyses (CEAs). For example, the cancer drugs carfilzomib and bortezomib were reported to have the highest rate of drug wastage (37% and 30%, respectively [2]), but while some CEAs include wastage in calculating drug cost, others do not mention drug wastage or vial sizes [25,26,27,28].

There are several limitations to our study. Our estimates of EV wastage were sensitive to the proportion of patients who receive this drug, and there is substantial uncertainty about this as a result of EV’s recent market entry. This was addressed by preforming a wide range sensitivity analysis (Table 2), incorporating both clinical trial and real-world data, as described in the methods section in detail. Local practices regarding utilization of the optimal composition of vials and rounding may vary and may potentially increase the proportion of wastage. The number of treatment doses per patient was based on MSKCC practice patterns, which may not accurately reflect the community setting. We accounted for such potential differences using a sensitivity analysis. We attempted to account for possible future trends in the treatment paradigm by estimating wastage in the 1st-line setting based on the EV-103 trial [8]. There were several uncertainties in estimating the number of patients who would be treated with EV in combination with pembrolizumab in the 1st line setting. First, the number of newly diagnosed metastatic UC patients per year was estimated, as the SEER database [12] and others numerate only de novo metastatic disease, but most patients with metastatic disease present with localized muscle-invasive disease progressing to metastatic disease. As metastatic UC is incurable, the number of UC deaths correlates to some extent to the number of patients diagnosed with metastatic disease a few years prior. As survival is slightly increased with modern treatments, we estimated that the number of newly diagnosed metastatic UC patients per year would be 20% more than the number UC deaths in 2020 per SEER database. Second, we estimated the percent of newly diagnosed metastatic UC patients who would receive this combination as 1st line treatment once approved, based on the clinical judgement of the authors, practicing genitourinary oncologists, as this treatment combination is suitable for cisplatin-eligible and cisplatin-ineligible patients and is well tolerated. These assumptions were addressed in the sensitivity analysis.

The major strength of our study is real-world data that may better represent the general population of advanced UC patients as compared with clinical trial participants.

## 5. Conclusions

EV wastage due to available vial sizes at a large tertiary hospital in 2020 was 2.9%, which falls under acceptable thresholds. Average waste cost per dose and per patient was $252 and $3127, respectively. Estimated annual costs of EV wastage in the US are between $6–44 million in the third line and may increase to $57–104 million if approved in first line.

## Figures and Tables

**Table 1:** Variables.

EV Dose and Cost	
Drug name	Enfortumab Vedotin
US FDA approved dose in mUC (in mg/kg)	1.25
Optional dose reduction dosing (in mg/kg)	1, 0.75, 0.5
Vial sizes available (in mg)	20, 30
EV cost, USD per mg	111.8
**EV-301 Data**	
Median number of doses per patient	16
**MSKCC Data for EV-Treated Patients in 2020**	
Number of patients	64
Number of doses administered	793
Median number of doses per patient	11 (IQR 6–18)
Estimated amount of drug used by optimal vial sizes, mg *	61,960
Actual amount of drug administered, mg	60,170
Estimated amount of drug wasted, mg	1790
Estimated % of drug wasted	2.9
Wastage cost, USD total	200,122
Wastage cost, USD/patient	3127
Wastage cost, USD/dose	252
**Without Rounding**	
Total drug per ideal dose, mg **	60,132
Estimated amount of drug wasted, mg	1828
Estimated % of drug wasted	3.0
Wastage cost, USD total	204,370
Wastage cost, USD/patient	3193
Wastage cost, USD/dose	257

* Based on total mg of drug available in optimal set of vials; ** Ideal dose defined as planned dose (mg/kg) × patient’s weight (kg). Abbreviations: EV, Enfortumab Vedotin; mUC, Metastatic Urothelial Cancer; MSKCC, Memorial Sloan Kettering Cancer Center.

**Table 2:** Sensitivity analysis.

Variable	Base Case Value	Lower Range	Upper Range	Note
Estimated number of UC cancer deaths in 2020	17,980	16,182	19,778	+/−10%
Percentage of metastatic UC patients receiving ≥3rd line treatment	30%	15%	50%	See Methods
Number of metastatic UC patients receiving EV as ≥3rd line treatment	5394	2427	9889	UC cancer deaths × % of eligibility
Wastage cost per dose, USD	252	227	277	+/−10%
Number of doses per patient	11	11	16	MSKCC: 11 EV-301 trial: 16
Estimated annual US cost of EV wastage for ≥3rd line treatment, USD	14,952,168	6,060,219	43,828,048	-

Abbreviations: EV, Enfortumab Vedotin; UC, Urothelial Cancer; MSKCC, Memorial Sloan Kettering Cancer Center.

**Table 3:** Structural sensitivity analysis of EV as 1st line.

Variable	Base Case Value	Lower Range	Upper Range	Note
Estimated newly diagnosed metastatic UC patients per year	21,576	19,418	23,734	+/−10%
Number of UC patients receiving 1st line EV + pembrolizumab	17,261	15,535	18,987	80% of annual metastatic UC patients
Wastage cost per dose, USD	252	227	277	+/−10%
Number of doses per patient	18	16	20	Per EV-103 trial +/−10%
Estimated annual US cost of EV wastage for 1st line treatment, USD	78,294,989	57,077,047	104,210,630	-

Abbreviations: EV, Enfortumab Vedotin; UC, Urothelial Cancer.

## Data Availability

The data presented in this study are available on request from the corresponding author.
